# Outcomes of total hip arthroplasty with a standard prosthesis for the affected hip in patients with poliomyelitis sequelae: a mid-term retrospective follow-up study

**DOI:** 10.1186/s13018-023-03697-6

**Published:** 2023-03-13

**Authors:** Lihan Shi, Guangwei Che, Yong Huang, Min Yi, Pengde Kang

**Affiliations:** 1 Department of Orthopaedics Surgery, Hospital of Chengdu University of Traditional Chinese Medicine, No. 39 Shi-er-qiao Road, Chengdu, 610036 Sichuan Province People’s Republic of China; 2 Department of Orthopaedics Surgery, West China Hospital, Sichuan University, 37# Wainan Guoxue Road, Chengdu, 610041 Sichuan Province People’s Republic of China

**Keywords:** Poliomyelitis sequelae, Standard prosthesis, Total hip arthroplasty, Complications, Outcomes, Prosthetic dislocation

## Abstract

**Introduction:**

Total hip arthroplasty for poliomyelitis sequelae could be a technical challenge due to the higher risk for prosthetic dislocation and degenerative changes in the affected limbs. This study aimed to analyse the mid-term outcomes of primary total hip arthroplasty on the affected hip with standard prosthesis.

**Materials and Methods:**

From January 2008 to January 2018, 32 patients with poliomyelitis sequelae underwent total hip arthroplasty on the affected hip with standard prosthesis. Clinical and radiographical outcomes, complications, and prosthesis survival rates were evaluated.

**Results:**

After a mean follow-up of 7.9 (4.4–13.1) years, the Harris Hip Score, University of California Los Angeles activity level rating, and 12-item Short Form Health Survey Questionnaire scale score significantly improved. The abduction and flexion motions of the hip joint improved dramatically, and the visual analogue scale pain score decreased significantly. The leg length discrepancy was effectively corrected. During the follow-up, one patient experienced prosthetic dislocation, one underwent revision surgery due to acetabular component loosening, two had osteolysis, four had heterotopic ossification, two experienced transient sciatic nerve palsy, and one had intermuscular vein thrombosis. The prosthesis survival rate was 96.9% at 5 years postoperatively. No periprosthetic infection occurred.

**Conclusion:**

Total hip arthroplasty with standard prosthesis could be an effective treatment for hip arthropathy on the affected hip of patients with poliomyelitis sequelae, resulting in good clinical outcomes and few complications. Constrained liner and dual mobility articulation are not recommended unless the hip muscle strength of the abductor is < III.

## Introduction

Poliomyelitis is a viral disease that is associated with sequelae in approximately 50% of patients [1], which includes varying degrees of flaccid paralysis, muscular atrophy, limb shortening, physiological hyporeflexia, and deformed skeletal development in the affected limb [2, 3]. Patients often have developmental dysplasia of the hip and osteoarthritis in the weight-bearing joint of the affected limb [4], especially the hip joint.

Total hip arthroplasty (THA) is an effective treatment for end-stage hip arthropathy; however, surgeons avoid using this treatment in patients with poliomyelitis sequelae, and some prefer using constrained liner and dual mobility articulation [5–8]. However, there are certain disadvantages of these prostheses, such as high cost, intraprosthetic dislocation, and a high rate of prosthetic loosening[9–11]. Therefore, the effectiveness of standard prostheses used for THA in patients with poliomyelitis sequelae is worth exploring. However, as hip arthropathy associated with poliomyelitis has become less common due to the popularisation of vaccines, there are limited studies on the outcomes after THA in these patients [12–17], while recent studies assessing the outcomes of THA using standard prosthesis in these patients are lacking. Therefore, this study evaluated mid-term clinical and radiographical outcomes, survivorship, and complications of patients with poliomyelitis sequelae who underwent THA with standard prosthesis.

## Materials and methods

Data of 56 patients (60 hips) with poliomyelitis sequelae who underwent THA from January 2008 to January 2018 due to severe pain and dysfunction that did not respond to the previous non-operative treatment were reviewed. After excluding seven patients (7 hips) undergoing THA on the nonparalyzed side, four patients (8 hips) undergoing bilateral THA, 11 patients (11 hips) followed up for less than two years, and two patients who died of cardiopulmonary disease during follow-up, which is not connected with the surgery,and these 2 patients did not have any revision operation until death.32 patients (62.5% women, 32 hips) were finally included. Of them, 25 patients (78.1%) had developmental dysplasia of the hip (DDH, including 20 patients with Crowe type I DDH, two with Crowe type II DDH, and three with Crowe type III DDH [18]), four (12.5%) had simple hip osteoarthritis (Tönnis grade 3 [19]), and three (9.4%) had femoral head necrosis secondary to femoral neck fracture (Association Research Circulation Osseous (ARCO) stage IV [20]). Surgical procedures were performed by the chief surgeons of the hospital. The study protocol was approved by the institutional review board and informed consent was obtained from all patients before surgery.

### Patient evaluation

Patients were evaluated preoperatively, at 1, 3, and 6 months postoperatively, and annually thereafter. Hip function was evaluated using the Harris Hip Score system. The activity level was evaluated using the University of California Los Angeles activity level rating (UCLA). The quality of life of patients was assessed using the 12-item Short Form Health Survey Questionnaire scale (SF-12). Hip joint pain was assessed using the visual analogue scale (VAS). The limb-length discrepancy (LLD) was assessed by measuring the length from the umbilicus to the bilateral medial malleoli using a measuring tape. The severity of limping was assessed using a four-point ordinal scale. The hip muscle strength was assessed using manual muscle testing (MMT). The hip flexion angle and abduction angle of the hip joint were measured using a protractor, and complications, including lower extremity deep vein thrombosis, periprosthetic infection, and prosthetic dislocation, were recorded until the end of the follow-up. The time until revision for any component due to any reason was recorded. All data were collected by the collaboration of two researchers (LHS and GWC).

Radiographic evaluation was conducted preoperatively, at 1, 3, and 6 months postoperatively, and annually thereafter. Acetabular cup inclination was calculated based on the method of Murray et al. [21], and the acetabular cup anteversion was calculated based on the method described by Lewinneck et al. [22]. The femoral component was diagnosed as loosened if it is progressively inverted or descended by less than 5 mm. The acetabular component was diagnosed as loosened if the position changed or if there were continuous lucent lines with a width of less than 0.2 mm [23]. Osteolysis around the acetabular prosthesis was described according to the criteria of DeLee et al. [24]. Osteolysis around the femoral prosthesis was assessed using the method described by Gruen et al. [25]. As described by Kim et al. [26], changes in the vertical distance between the greater trochanter and the lateral margin of the femoral stem and the vertical distance between the medial margin of the femoral stem and the lesser trochanter were measured to determine whether the stem was sinking. Based on the initial postoperative X-ray measurements, it could be determined as prosthetic sinking if the measurement difference was 3 mm or higher. Heterotopic ossification was evaluated using the Brooker grading system [27].

### Perioperative management and surgical methods

THA was performed by five senior surgeons (PDK, ZKZ, JY, BS, and FXP) using a posterolateral approach, with the patients in a lateral position. Abductor muscle exercises were started 3 months preoperatively. Surgical procedures were performed as described previously in detail by Barrett et al. [28]. The posterior joint capsule and external rotators were carefully repaired after reduction, and standard prostheses were used in all cases. All acetabular components were from PINNACLE® (DePuy, Warsaw, IN, USA), and all femoral components were from Corail® Stem (DePuy, Warsaw, IN, USA). The bearing surface was ceramic-on-ceramic in 11 hips (34.4%), ceramic-on-polyethene in 20 hips (62.5%), and ceramic-on-metal in one hip (3.1%). The acetabular component was implanted at anteversion angles of 15–25° and inclination angles of 40–50°. One or two screws were used in the acetabular components to augment the fixation in nine hips (28.1%). An intraoperative proximal femoral fracture occurred in seven hips (21.9%), which was managed with cerclage wires. On postoperative day 1, all patients were encouraged to start performing hip abductor and flexor strengthening exercises in or out of bed.

### Statistical analysis

Statistical analysis was performed using SPSS 23.0 (IBM, Armonk, NY, USA). The results are expressed as mean ± standard deviation. Clinical outcomes were compared by repeated ANOVA or paired sample t-test. Kaplan–Meier survival analysis was used to assess the time until revision for any component. Statistical significance was set at p < 0.05.

## Results

### Clinical outcomes

The mean age of patients who underwent surgery was 54.7 (37–70) years. Primary wound healing was achieved in all patients after surgery. The mean follow-up time was 7.9 (4.4–13.1) years. Detailed operative characteristics are shown in Table 1.

**Table 1:** Demographics

Characteristic	Value
Number of patients (n)	32
Number of females (n)	20 (62.5%)
Number of males (n)	12 (32.5%)
Mean operation age (y)	54.7 (Range:37–70)
Mean follow-up time(y)	7.9 (Range:4.4–13.1)
Mean operation time(min)	59.8 (Range:40–115)
The mean intraoperative blood loss(ml)	325.3 (Range:260–450 mL)
Mean hospital stay(d)	11.1 (Range:9–21)
Hip muscle strength	
Abductor	III (27);IV (5);V (0)
Extensor	III (17); IV (15);V (0)
Adductor	III (0);IV (11);V (21)
Flexor	III (0);IV (7);V (25)

By the final follow-up, the abduction and flexion angles of the affected hip increased markedly, Harris hip score improved, the rate of excellent and good condition was 75%, physical component summary and mental component summary based on SF-12 scores increased, UCLA score improved, VAS score decreased remarkably, and LLD decreased significantly (all p < 0.001) (Table 2), and 25 patients had complete pain relief in the operated hip (the remaining patients experienced mild pain after walking for more than 1 h). Ninet

**Table 2:** Clinical outcomes

Indicator	Mean and Standard Deviation				*P* value						
	Preoperative	1 year postoperative	3 years postoperative	final follow-up	I	II	III	IV	V	V	V
Harris Hip Score(point) ^a^	56.66 ± 8.70	75.59 ± 5.11	79.25 ± 5.07	83.53 ± 4.36	< 0.01	< 0.01	< 0.01	< 0.01	< 0.010.095	< 0.01	< 0.01
UCLA activity-level rating(point) ^a^	3.69 ± 0.97	5.56 ± 0.56	5.88 ± 0.66	6.22 ± 0.66	< 0.01	< 0.01	< 0.01	< 0.01	< 0.01	< 0.01	< 0.01
SF-12^a^											
PCS (point)	30.47 ± 6.33	47.56 ± 4.51	51.47 ± 4.13	54.38 ± 3.99	< 0.01	< 0.01	< 0.01	< 0.01	< 0.01	< 0.01	< 0.01
MCS (point)	45.66 ± 5.10	52.94 ± 3.47	55.00 ± 3.36	56.13 ± 3.28	< 0.01	< 0.01	< 0.01	< 0.01	< 0.01	< 0.01	< 0.01
Visual Analogue Score(point) ^a^	6.19 ± 0.90	1.81 ± 0.47	0.31 ± 0.64	0.22 ± 0.42	< 0.01	< 0.01	< 0.01	< 0.01	< 0.01	< 0.01	0.499
Hip flexion(°) ^a^	64.84 ± 9.80	88.75 ± 6.96	91.56 ± 6.15	93.13 ± 6.19	< 0.01	< 0.01	< 0.01	< 0.01	0.017	< 0.01	0.095
Hip abduction(°) ^a^	22.81 ± 5.23	30.47 ± 3.45	31.72 ± 3.73	32.97 ± 4.19	< 0.01	< 0.01	< 0.01	< 0.01	0.11	0.008	0.181
Limb length discrepancy(cm) ^b^	3.11 ± 0.74	–	–	1.10 ± 0.42	< 0.01						

I, Preoperative versus 1 year postoperative versus 3 years postoperative vs final follow-up; II, Preoperative versus 1 year postoperative; III, Preoperative versus 3 years postoperative; IV, Preoperative versus final follow-up; V, 1 year postoperative versus 3 years postoperative; VI, 1 year postoperative versus final follow-up; VII, 3 years postoperative vs final follow-up,

^a^P values were calculated by repeated ANOVA

^b^P values were calculated by paired aample T test

een patients still had claudication at the final follow-up, of which four had moderate claudication, 15 had mild claudication, and none had severe claudication.

### Radiographical outcomes

**Fig. 1:**
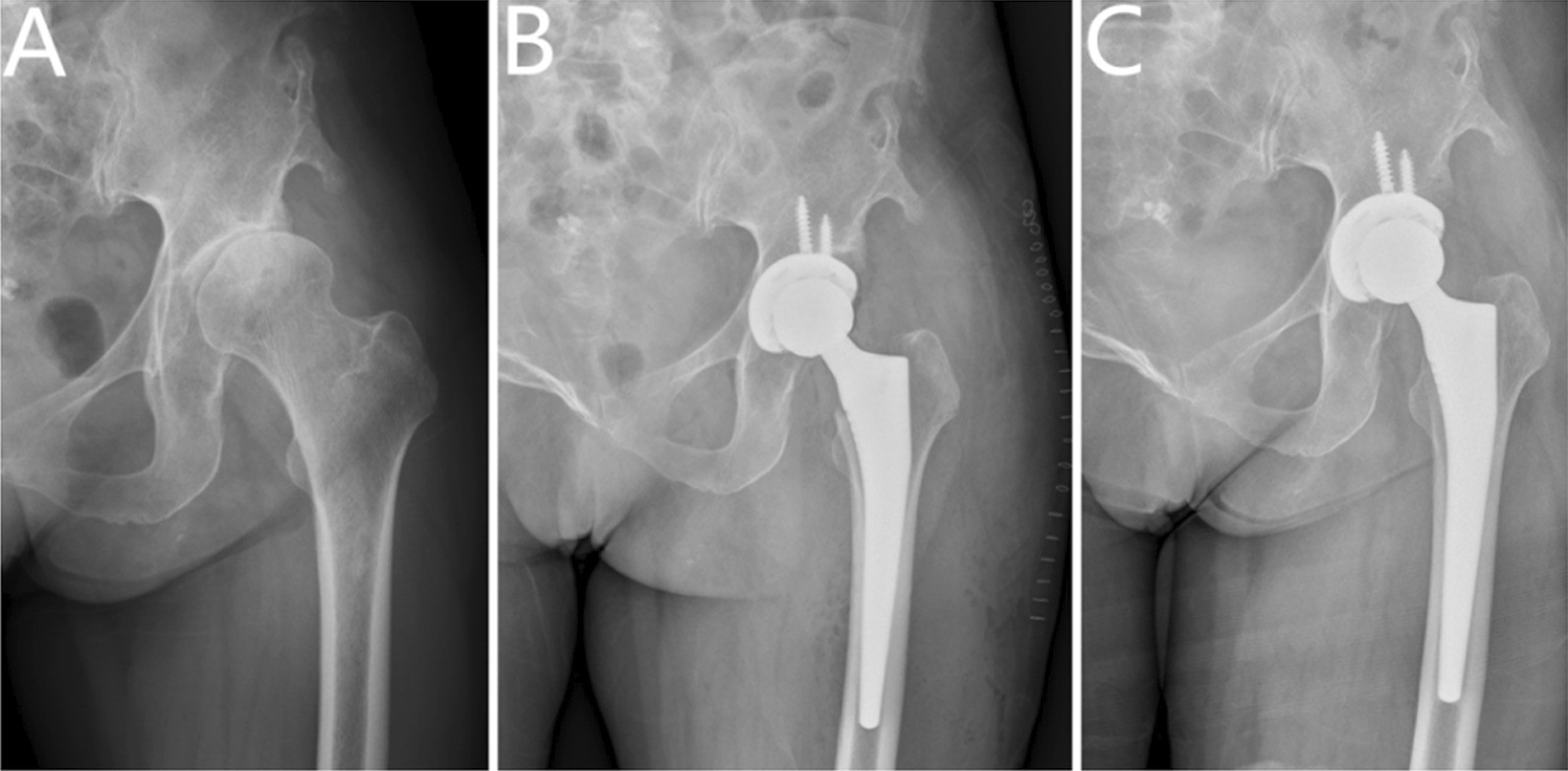
**A** Preoperative radiograph of the hip of a 56-year-old female with residual poliomyelitis,showing a osteoarthritis secondary to developmental dysplasia of the hip. **B** postoperative imaging. **C **Radiograph obtained 4.7 years after surgery,showing no evidence of implant subsidence,implant loosening or osteolysis

The acetabular cup anteversion angle was 18.72 (13–23)°, with an inclination angle of 43.19 (40–49)°, all of which were within a safe range. One patient had progressive lucent lines with a width of > 0.2 mm in zones II and III of the acetabulum, which caused acetabular cup loosening. This patient underwent revision surgery 4.5 years after the surgery. At the end of the follow-up, no patient had signs of loosened components or subsidence of the femoral stem, one patient had a lucent line of < 1 mm in the proximal femur at the 7-year follow-up after the surgery, located in the Gruen I and VII zones, one patient had speckled osteolysis around the acetabulum at a follow-up at 5 years after surgery, located in zone III. During the follow-up period, heterotopic ossification was found on the radiographs of four patients and based on the Brooker grading system [27], three patients were of grade I and one was of grade II (Fig. 1; Table 2).

### Complications

**Fig. 2:**
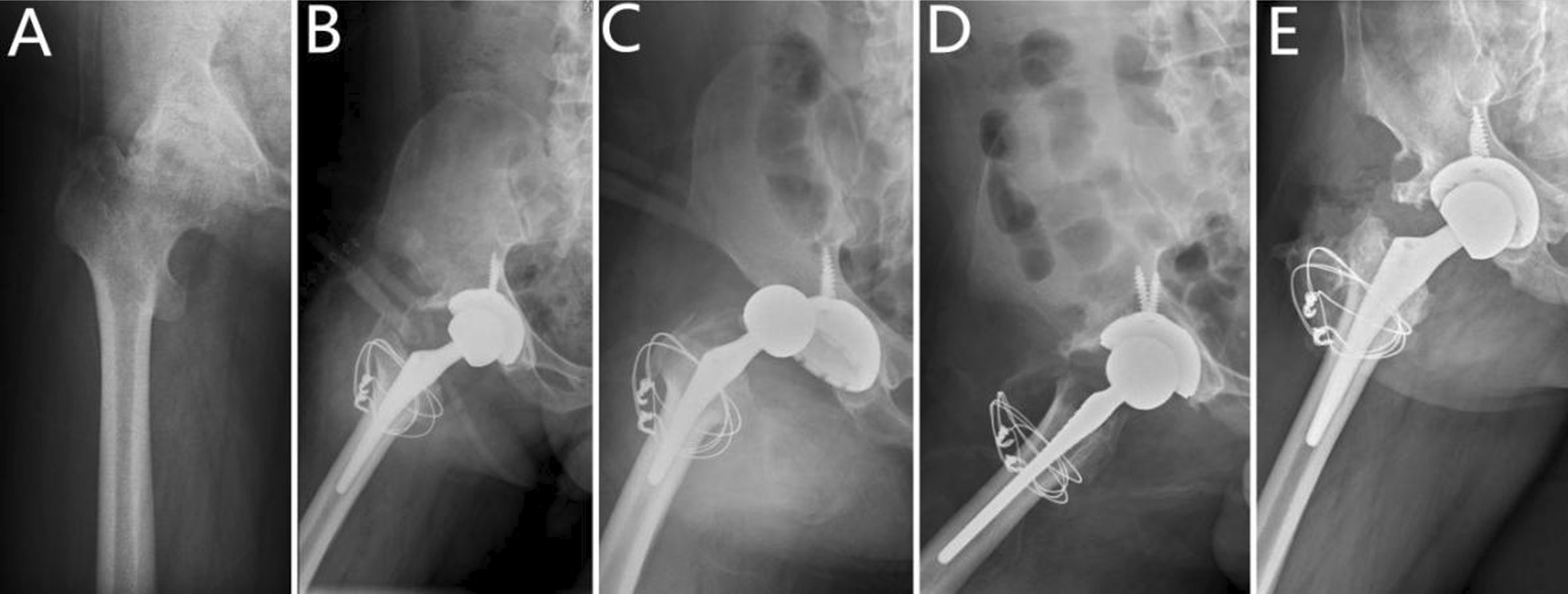
**A** Preoperative radiograph of the hip of a 43-year-old male with residual poliomyelitis,showing a osteoarthritis secondary to developmental dysplasia of the hip. **B** Postoperative radiograph. **C**Radiograph obtained 1 month after surgery showed dislocation of the hip. **D** Radiograph obtained after the manual replacement on the dislocation of the hip. **E** At 5.4-year follow-up,radiograph showed a classII heterotopic ossification(Brooker classification system),no evidence of implant subsidence, implant loosening or osteolysis, no dislocation recurred

Intraoperative proximal femoral fracture occurred in seven hips, while two patients developed transient sciatic nerve palsy due to surgical limb extension and completely recovered approximately 6 and 8 months after the surgery, respectively. One patient had a prosthetic dislocation because of an accidental fall 1 month after the surgery, after manual reduction and lower extremity abduction brace fixation for 6 weeks, there was no further dislocation at the final follow-up (Fig. 2). One patient had intermuscular vein thrombosis after the surgery, and the lower extremity vascular colour Doppler ultrasound showed that the thrombosis disappeared by the end of the follow-up following thrombolytic therapy and no periprosthetic infections, fractures, or other complications occurred.

### Survival rate analysis

**Fig. 3:**
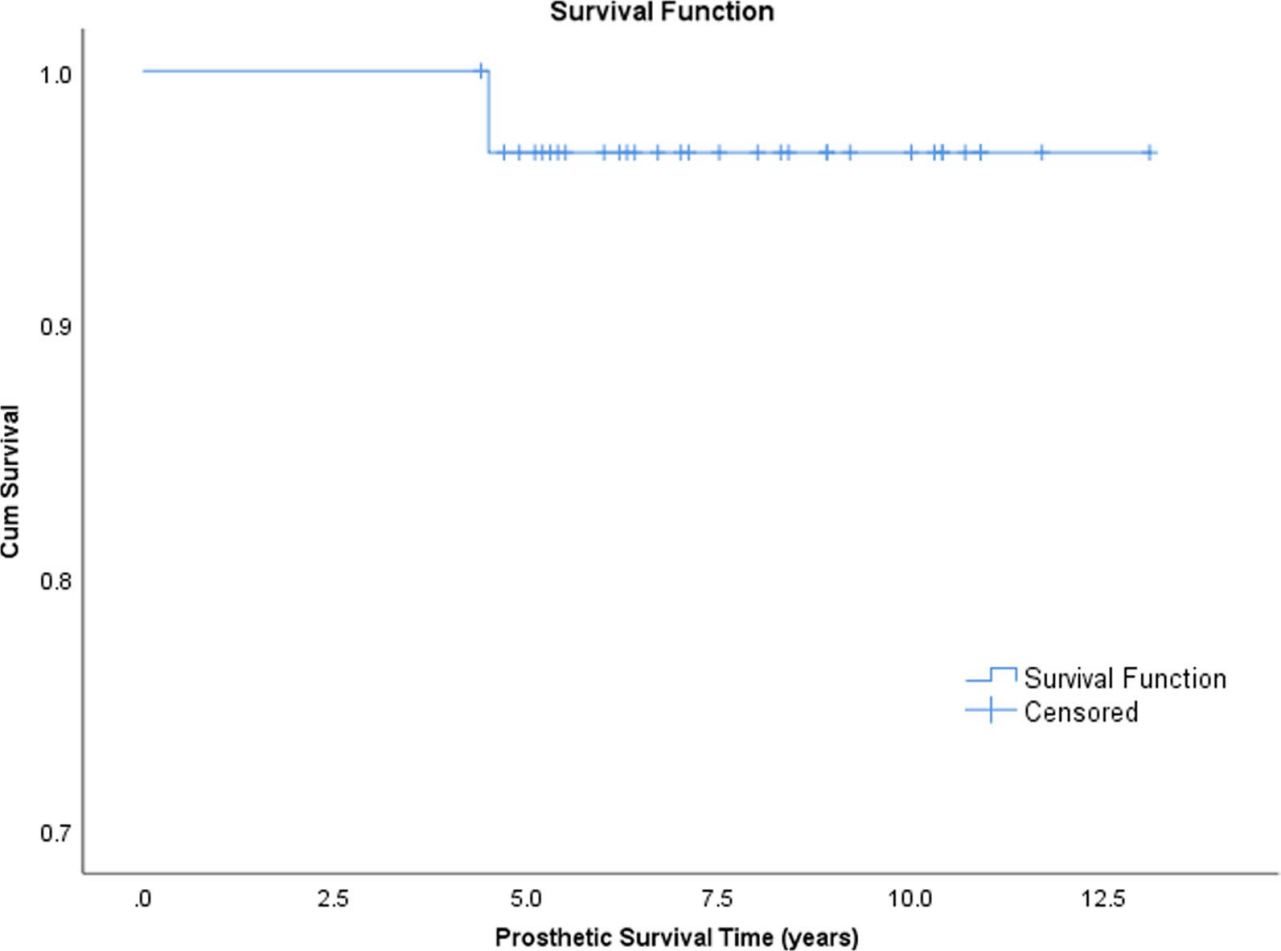
Kaplan–Meier analysis of time without revision for any reason for any component

At 5 years after surgery, the survival rate was 96.9% (95% confidence interval 83.8–99.9%), including any revision surgery due to any reason set by the study endpoints (Fig. 3).

## Discussion

To our knowledge, this is the largest clinical follow-up study in China on patients with poliomyelitis receiving standard prosthetic THA in the affected hip. The outcomes were satisfactory over a mean follow-up period of 7.9 years. Patients’ mobility and quality of life were greatly improved. These findings strengthen the existing evidence that THA can be effective in treating hip arthropathy in patients with poliomyelitis sequelae with minimal complications.

Patients with poliomyelitis sequelae have paralysis of the hip abductors and extensors in the affected limb, while the adductors and flexors are essentially normal, resulting in a higher risk for artificial hip dislocation after THA. Therefore, many surgeons adopt a more conservative approach in these patients, which is inadequate for end-stage hip disease. Some scholars recommended the use of a restrictive liner to reduce the risk of dislocation [29, 30]; however, there is still a risk of acetabular cup loosening in the long term. Spinnickie et al. [29] reported a dislodgment of the artificial femoral head, which was still located within the restrictive liner, in a 71-year-old elderly male with poliomyelitis sequelae who underwent THA with a restrictive liner for an intertrochanteric fracture; revision surgery was performed using a large diameter femoral head and unconstrained liner to increase stability. A systematic review by Gaurav et al. [31] also found good results with the use of unconstrained acetabular components compared to constrained implants in THA in polio patients with abductors and gluteal muscle paralysis. Also, dual mobility hip implants had been used to reduce the risk for dislocation in THA in polio patients [8]. However, the dual mobility cup system is more expensive than a standard prosthesis, with a 0.7–4% incidence of internal dislocation [32, 33].

In the scheduled return visits of clinical outcomes, we observed that the Harris score, UCLA mobility score, and SF-12 score of the patient group significantly improved, and the VAS score significantly decreased. Also, most patients experienced an improvement in the function, mobility of the affected hip joint and quality of life. Specifically, the pain of the affected hip joint was effectively relieved, although seven patients still felt slight pain after walking for more than 1 h, which could be relieved after rest. This situation might be related to the high activity level of these patients. By recommending appropriate physical activity at follow-up, the situation improved in most patients. Nineteen patients still had varying degrees of limp, and one of them required a cane to walk for a long time; we speculate that this was caused by the pain of his affected hip joint and the unequal length of his lower limbs. Most patients were satisfied with the surgical outcome because the surgery significantly relieved the hip pain, as most were older and relatively inactive, and mild to moderate limping was acceptable. Two patients developed transient postoperative sciatic nerve palsy, which might be related to their intraoperative limb lengthening of more than 3 cm; however, they fully recovered after pharmacological treatment at 6 and 8 months after surgery, respectively.

Follow-up imaging showed various degrees of periprosthetic osteolysis in three patients (9.4%). This rate was substantially lower than the rate of 15.6% that was reported in another study [14]. One of these three patients showed prosthetic loosening and underwent a revision surgery at 4.5 years postoperatively. By the end of the follow-up, no prosthetic loosening had occurred, and the prosthetic survival rate had reached 96.9%, which we considered satisfactory. Ectopic ossification occurred in four patients (12.5%), which may be related to the more extensive intraoperative soft tissue dissection in these patients, and the incidence was slightly lower than that reported by DeDeugd et al. [14] (19%). One patient had prosthetic joint dislocation 1 month postoperatively and underwent manual reduction, which may have exacerbated the soft tissue injury, causing heterotopic ossification. In addition, femoral split fracture occurred in seven patients during the intraoperative installation of the femoral stem. The study of Argenson et al. [34] suggested that the higher intraoperative incidence of the femoral split fracture might be due to the narrow femoral medullary cavity in patients with developmental hip dysplasia,making a longitudinal slot on the femoral cortex with a saw and prophylactic wire cerclage could be a good choice to prevent the femoral split fracture,which was described by Wu[35],In this group of patients, we recommend longer periods of bed rest with plyometric training.

Patients with the late-onset sequelae of poliomyelitis are prone to hip dysplasia on the affected side, which may be related to the stronger adductor and flexor muscles than the paralyzed abductor and extensor muscles of the affected hip, which in turn gradually leads to dysplasia of the acetabulum [36, 37] and higher risk for postoperative artificial joint dislocation. In our study, 25 patients (78.1%) had hip dysplasia. Recent studies[4, 12–17, 38, 39] show a dislocation rate of 7.8% in THA using standard prosthesis in patients with poliomyelitis sequelae(Table 3), which is higher than that of patients without any neuromuscular diseases who underwent primary THA (2–3%) [40], and broadly in line with the dislocation rate following THA in patients with other neuromuscular diseases (6%) [5]. However, the dislocation rate in our study series was 3.1%, which was lower than the average level of such patients and was largely consistent with the dislocation rate in patients without any neuromuscular disease. This is a highly encouraging result, with only one patient in our study experiencing an artificial hip dislocation due to an accidental fall 1 month postoperatively. The patient underwent manual reduction under general anaesthesia, followed by an abduction brace for fixation for 6 weeks, and after removal of the brace, he was repeatedly instructed to strengthen the abductor muscles of the affected hip, and no further dislocation had occurred by the end of the follow-up.

**Table 3:** Summary of available reports of THA with a standard prosthesis in patients with residual poliomyelitis

Author	Hips(n)	Year	Mean follow-up(year)	Prosthesis	Dislocation, N (%)
Sonohata[4] (Japan)	1	2016	6.0	Standard	0 (0%)
Yoon[12] (South Korea)	4	2014	7.4	Standard	0 (0%)
Faldini[13] (Italy)	14	2017	7.7	Standard	0 (0%)
Dedeugd[14] (USA)	32	2018	6.0	Standard	3 (9.4%)
Sonekatsu[15] (Japan)	2	2018	8.4	Standard	0 (0%)
Cho[16] (South Korea)	4	2016	7.0	Standard	1 (25%)
Buttaro[17] (Argentina)	6	2017	10.0	Standard	1 (16.7%)
Wicart[38] (France)	2	1999	5.0	Standard	1 (50%)
Sobrón[39] (Spain)	5	2017	4.6	Standard	1 (20%)
Our study (China)	32	2022	7.9	Standard	1 (3.1%)
Total	102				8 (7.8%)

The low dislocation rate in our study may be due to the great emphasis on abductor muscle strength training in our patients; we encourage our patients to perform adductor muscle strength training of the affected hip 3 months before admission so that their abductor muscle strength is above grade III on admission (Table 1). In addition, the seven patients who had intraoperative femoral split fracture were instructed to stay in bed for abductor and flexor muscle strength training on the first postoperative day, and their ambulation training time was delayed for 6–8 weeks. The remaining 23 patients were encouraged to start ambulation training on the first postoperative day, to continuously perform it after discharge, and to have a regular outpatient follow-up. Some studies have shown that the incidence of dislocation in THA via the posterior approach was 5.8%, while that of the anterior approach was 2.3%. This may be due to the fact that the postero-lateral approach requires compromising of the posterior external rotator muscle group and the posterior joint capsule, possibly resulting in posterior soft tissue weakness and posterior dislocation. All our patients underwent the procedure via the postero-lateral approach, and intraoperative imaging determined that the anteversion and abduction angles of the acetabular prosthesis were within the normal range.The posterior joint capsule and external rotation muscle group were carefully repaired, which may be another important reason for the lower dislocation rate in our study,and another recent study,which focus on outcomes of total hip arthroplasty in juvenile patients,had similar view [41].

Our study had some limitations. First, due to the promotion of vaccines, the number of eligible patients is gradually decreasing, which is unavoidable. Second, there was no corresponding control group as a reference. Third, due to the limited number of patients, only a retrospective study could be performed. Finally, LLD was measured using a measuring tape, which was not as accurate as imaging measurements. However, to the best of our knowledge, this is the first consecutive case series report on the outcomes of THA with a standard prosthesis for the affected hip of patients with poliomyelitis sequelae in China, and although the length of the follow-up period was not uniform for all patients, the mean follow-up time of 7.9 years was sufficient to evaluate the clinical efficacy in such patients.

In conclusion, THA with a standard prosthesis for the affected hip can achieve good outcomes in patients with poliomyelitis sequelae, such as the improvement of the hip joint function, patient mobility and quality of life, as well as the high stability of components with few complications. Given its unavoidable design disadvantages, we do not recommend using restrictive and dual-mobility prostheses in THA in patients with poliomyelitis sequelae unless the patient's abductor muscle strength of the affected hip could not maintain above grade III.

## Data Availability

This article contains data sets that support the conclusions of this article. All data are completely available and unlimited.
